# 

**Published:** 1890-05

**Authors:** 


CONTENTS:

PAGE

ORIGINAL ARTICLES:
Review of "Contagious Venereal Disease Amongst Horses in Kent County, Canada." By W. L. Williams, V.S. . . . 261
Antiseptic Surgery. By E. T. Lawley-York . . . 267
Practical Hints. By Dr George F. Bower. . . . 276
Age of the Horse, Ox, Dog, and other Domestic Animals. By R. S. Huidekoper, M.D., Veterinarian . . . 279
EDITORIAL DEPARTMENT:
United States Veterinary Medical Association. . . . 289
SOCIETY PROCEEDINGS:
Long Island Veterinary Society. . . . 292
Veterinary Medical Association of New Jersey . . . 298
Veterinary Medical Society of Ontario College . . . 299
The Keystone Veterinary Medical Association. . . . 300
Veterinary Medical Society of the University of Pennsylvania . . . 301
Montreal Veterinary Medical Association. . . . 301
VETERINARY COLLEGE NOTES:
Ontario Veterinary College, . . . 302
Faculty of Comparative Medicine and Veterinary Science, McGill University, Montreal . . . 306
Chicago Veterinary College . . . 312
REVIEW DEPARTMENT:
Evolution and Disease. By Bland Sutton. . . . 313
A Practical Guide to Meat Inspection. By Thomas Walley, M.R.C.V.S. . 315
BOOKS AND PAMPHLETS RECEIVED. . . . 315

